# Supplementary figures and images for: Bioinformatics analysis of oxidative stress genes in the pathogenesis of ulcerative colitis based on a competing endogenous RNA regulatory network
Source: PeerJ. 2024 Aug 16;12:e17213. doi: 10.7717/peerj.17213 (PMC11332386; doi:10.7717/peerj.17213)

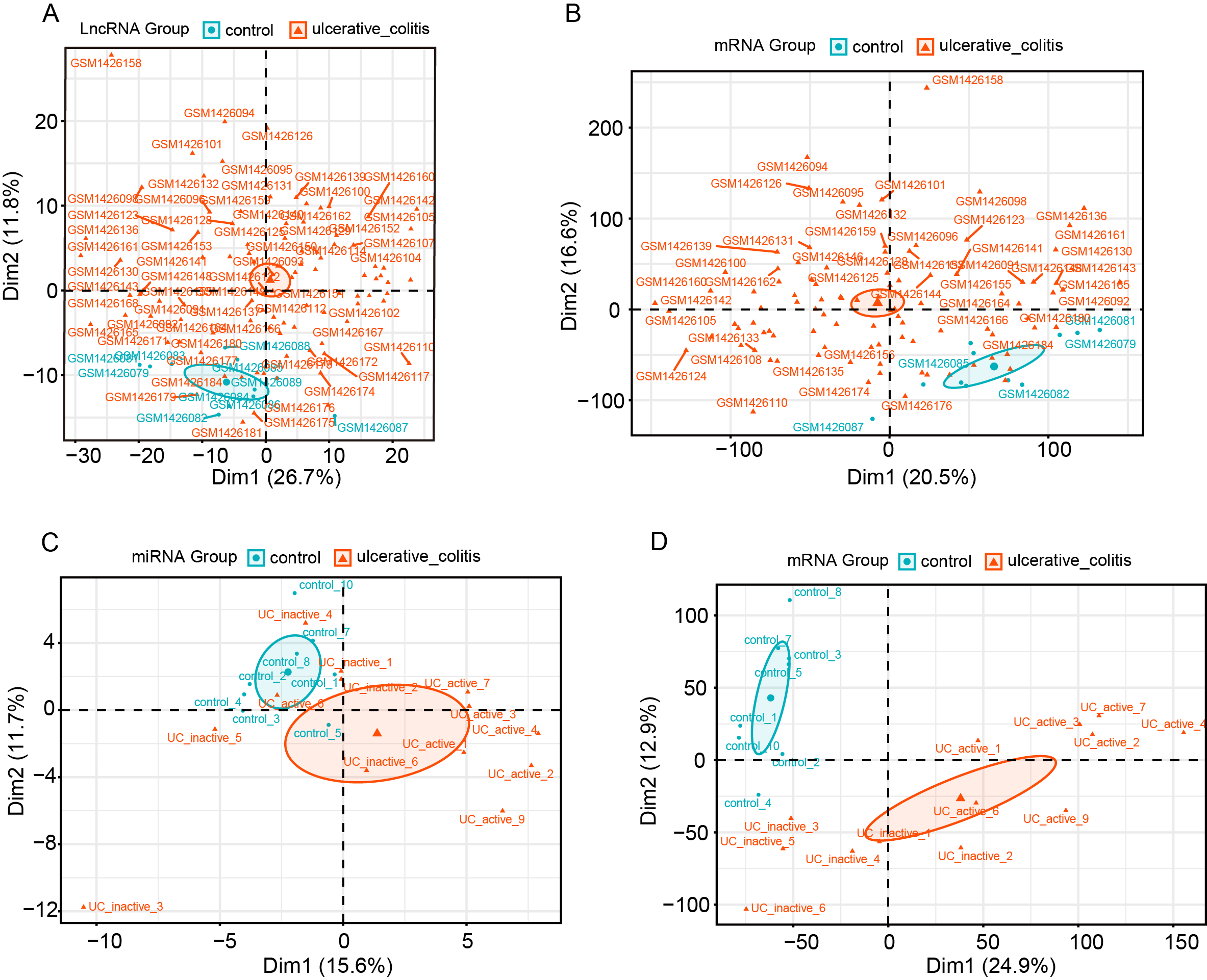

Supplement: Supplemental Information 3 — (A) Principal components analysis for lncRNA between control and UC samples in GSE75214 dataset. (B) Principal components analysis for miRNA between control and UC samples in GSE75214 dataset. (C) Principal components analysis for miRNA between control and UC samples in GSE48959 dataset. (D) Principal components analysis for mRNA between control and UC samples in GSE48959 dataset. [file peerj-12-17213-s003.png]

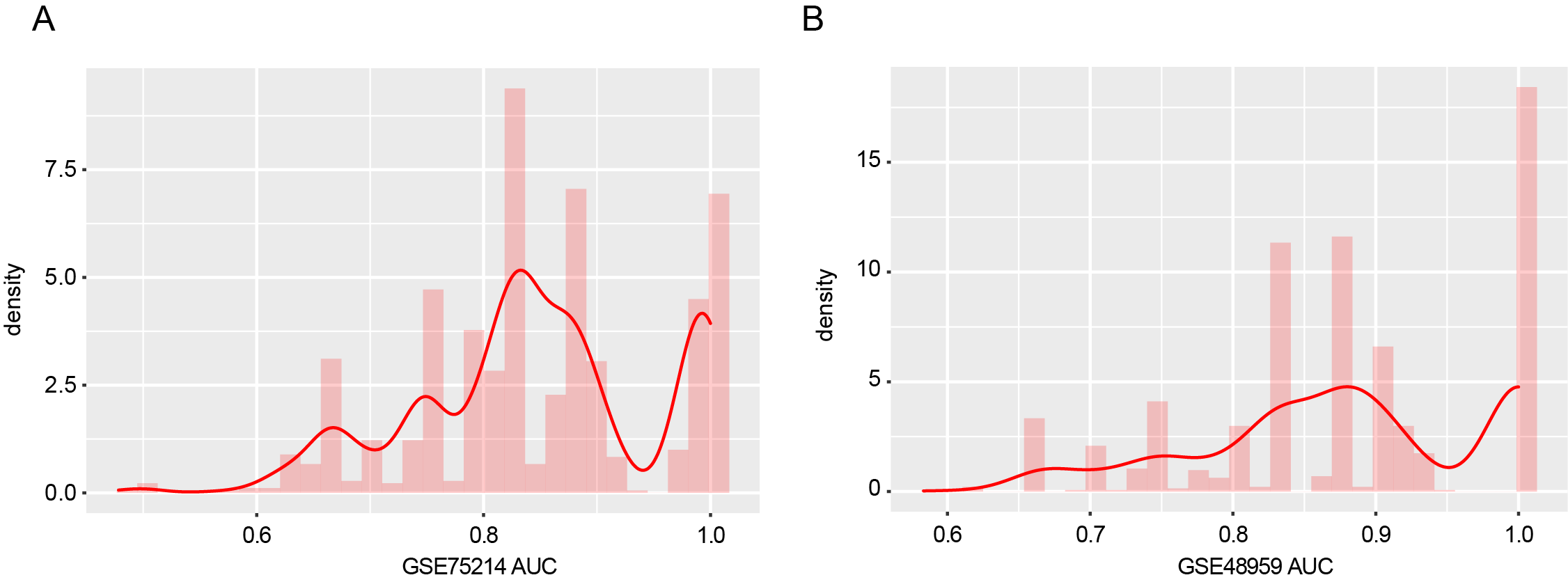

Supplement: Supplemental Information 4 — AUCand accuracydensitydistributions of GSE75214 (A) and GSE48959 (B) datasets. [file peerj-12-17213-s004.png]

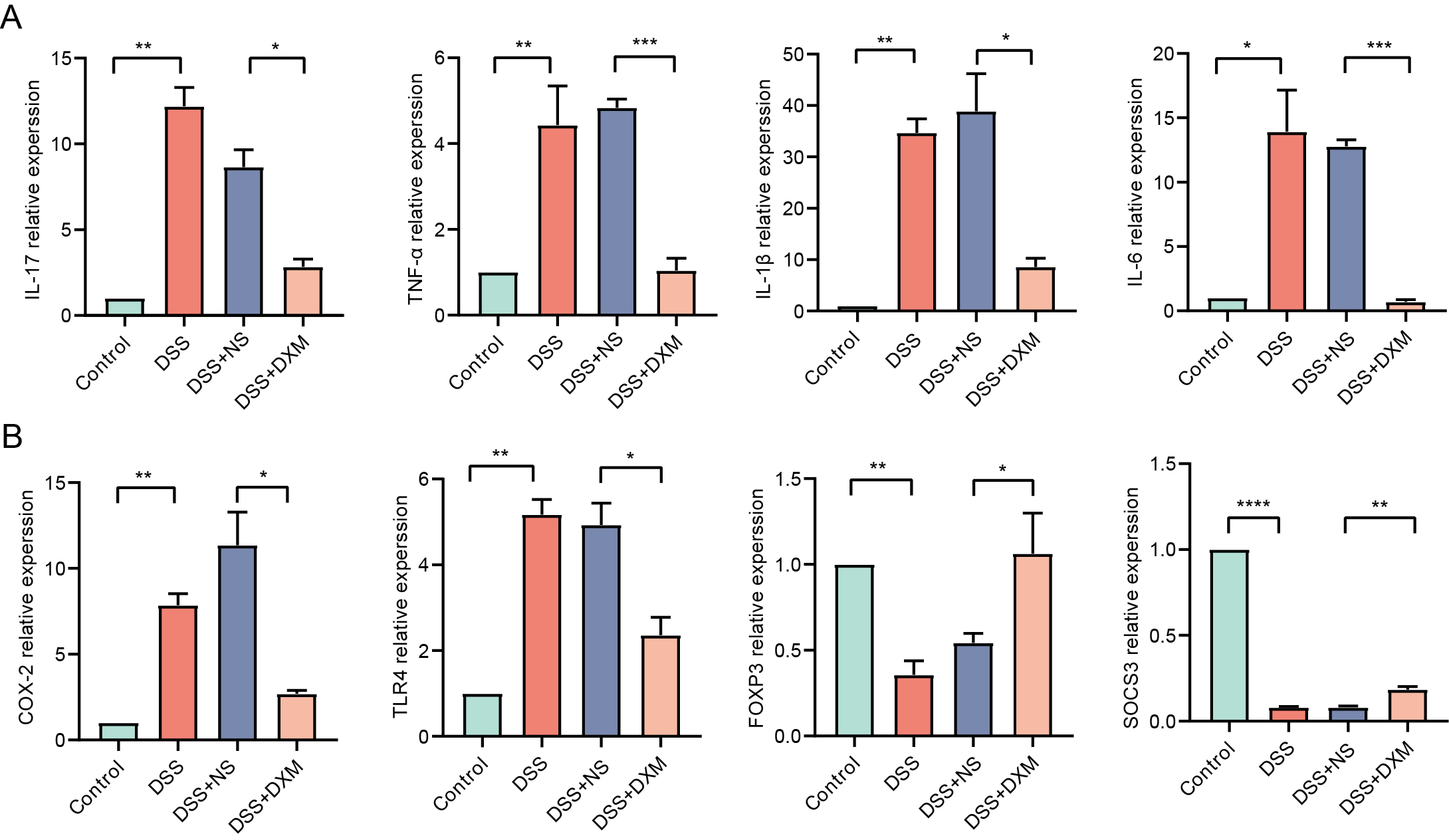

Supplement: Supplemental Information 5 — (A) The mRNA expression of inflammatory cytokines IL-17, TNF-α, IL-1β and IL-6 in different groups. (B) The mRNA expression of pro-inflammatory effector factors (COX-2 and TLR4) and anti-inflammatory effector factors (Foxp3 and SOCS3) in different groups. *P < 0.05, **P < 0.01, ***P < 0.001, ****P < 0.0001. [file peerj-12-17213-s005.png]
